# Perceptions of a longitudinal standardized patient experience by standardized patients, medical students, and faculty

**DOI:** 10.1080/10872981.2018.1548244

**Published:** 2018-12-04

**Authors:** Lauren Block, Judith Brenner, Joseph Conigliaro, Renee Pekmezaris, Barbara DeVoe, Andrzej Kozikowski

**Affiliations:** a Department of Medicine and Science Education, Donald and Barbara Zucker School of Medicine at Hofstra/Northwell, Hempstead, NY, USA; b Department of Science Education, Donald and Barbara Zucker School of Medicine at Hofstra/Northwell, Hempstead, NY, USA; c Department of Medicine, Donald and Barbara Zucker School of Medicine at Hofstra/Northwell, Hempstead, NY, USA; d Department of Medicine, Northwell Health, Center for Health Innovations & Outcomes Research, Manhasset, NY, USA; e Hofstra Northwell School of Graduate Nursing and Physician Assistant Studies, Hofstra University, Hempstead, NY, USA

**Keywords:** Standardized patients, clinical skills, self-efficacy, continuity, qualitative research

## Abstract

**Background**: Longitudinal standardized patient (LSP) experiences mimic clinical practice by allowing students to interact with standardized patients (SPs) over time. LSP cases facilitate practice, assessment, and feedback in clinical skills and foster an appreciation for the continuum of care.

**Objective**: We sought to characterize the nature of relationship-building, feedback, and continuity among all stakeholders participating in a single LSP program.

**Design**: We developed and implemented a novel LSP program. Students encountered two LSP characters six times each during the first 2 years of medical school, though continuity pairings of students, SPs, and faculty were frequently not possible. Focus groups were held with second-year medical students (*N* = 15), core faculty who coached these students in LSP encounters (*N* = 8), and SPs who had played the role of either LSP character (*N* = 10) participated. Results were analyzed thematically using a template analysis approach.

**Results**: The longitudinal nature of the experience reinforced the importance of student growth over time, the key role of faculty and SPs in providing feedback, and the tension between feedback and assessment. Students reported that LSP cases encouraged practice and feedback. SPs felt wedded to the longitudinal characters. Continuity pairings were recommended by all stakeholders to increase authenticity and promote relationship-building.

**Conclusion**: Stakeholders observed that the LSP cases brought some sense of continuity missing in other clinical skills encounters which helped prepare students for patient care. Continuity pairings of students, faculty, and SPs were recommended to enhance relationship-building and feedback.

## Introduction

Providing continuity of care and managing chronic disease are outlined as core skills for trainees by the American College of Graduate Medical Education and Liaison Committee on Medical Education [1,2]. According to the American Association of Medical Colleges national survey, 94% of US medical schools use standardized patient (SP) experiences [3]. Longitudinal standardized patient (LSP) experiences involve a medical student interacting with the same ‘patient’ over time in various scenarios. LSP experiences have been described as a means to provide structured opportunities for practice, assessment, and feedback in clinical skills and foster an appreciation for the continuum of care [4–6]. These interactions are designed to provide students with clinical experiences as well as individualized, developmentally sequenced assessment of clinical skills [7]. Students and faculty participating in a four-encounter LSP program in the Netherlands rated their program favorably on the authenticity of the experience and similarity of LSPs to real patients [8]. One study describing the integration of an LSP program into a family medicine clerkship reported increased student confidence in managing chronic disease and establishing relationships [9]. SPs described the approach as one which facilitates skill development through immediate feedback and performance adjustment [10]. However, the nature of relationship-building, feedback, and continuity in an LSP program has not been characterized among all stakeholders participating in a single program. This research is important as it would allow for a more comprehensive understanding of an LSP program.

We used a qualitative, template analysis approach to understand how a novel 2-year LSP experience, incorporating six visits with each of two characters, impacted the medical students, faculty, and SPs who participate in the program. Based on prior literature [4–6,9], the domains of continuity, relationship-building, and feedback were used as the initial model around which to build our understanding of the program.

## Methods

### LSP program

The Hofstra Northwell School of Medicine curriculum incorporated two standardized cases as part of a longitudinal clinical skills experience for first- and second-year medical students. Actors who portrayed these longitudinal patients were experienced SPs at the health system’s corporate training center. A total of 40 SPs (21 men and 19 women) participated in the LSP program. On average, SPs participated in four of the six visits for the LSP character. These SPs also played distinct characters in other medical student encounters at the clinical skills center. SPs undergo approximately 6 h of training in preparation for their role in medical student assessment [11,12].

Students encountered each of these two ‘LSPs’ six times during the first 2 years of medical school alongside approximately 21 other stand-alone SP cases. Of these 33 cases, 10 were summative and the remainder were formative. The first case follows a male patient struggling with alcohol abuse who developed gastrointestinal (GI) bleeding, gout, liver dysfunction, and atrial fibrillation. The second case is a female patient first encountered when pregnant, who delivers and raises a child with Down syndrome. Specific topics included in the LSP cases, chief complaint, and student skills assessed are shown in Table 1. Wherever possible, students were paired with the same SPs and faculty for multiple encounters, but due to conflicting schedules, continuity pairings were infrequent.

**Table 1.** Topics covered in longitudinal SP case.

Topic	Student skill	Case
*Screening for and counseling around alcohol abuse	Alcohol history	Larry
Smoking cessation	Motivational interviewing	Larry
Depression	Patient health questionnaire 9	Larry
*Gastritis	Social history	Larry
Gout	Interpreting diagnostic tests	Larry
Cholecystitis	Hypothesis-driven physical exam	Larry
GI bleeding	Explanatory model; oral presentation	Larry
Atrial fibrillation	Medication adherence	Larry
*Prenatal testing	Screening vs. diagnostic tests	Linda
*Down syndrome	Sharing emotionally challenging news	Linda
Vaccination counseling	Patient education	Linda
*Post-partum visit	Gynecologic history	Linda
Health Information Portability and Accountability Act (HIPAA) and privacy	Nondisclosure of protected health information	Linda
*Back pain	Eliciting an HPI	Linda

*Faculty-observed debriefs. Several encounters included more than one topic.

**Figure 1.**
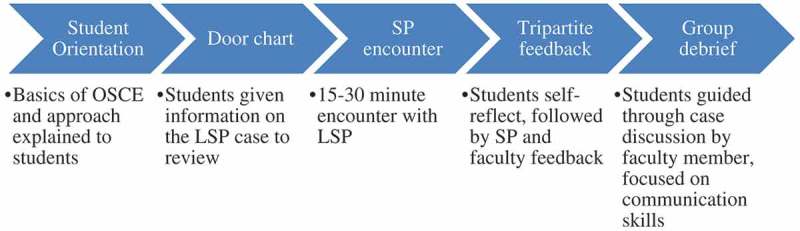
Longitudinal standardized patient encounters were conducted using a standardized format to reorient students to the case, permit adequate time for the encounter and feedback session involving SPs and faculty if present, and debrief the case in a group setting.

LSP case workflow is shown in Figure 1. Encounters lasted for approximately 20–40 min and were immediately followed by feedback by the SP. SPs provided feedback using a student-led, appreciative inquiry model initiating with positive aspects of student performance [13]. SPs remain ‘in character’ for these feedback sessions, keeping the name, symptoms, and personality traits of the character while offering feedback from the patient perspective. Following five of the encounters involving key communication or patient care skills, faculty evaluated students. Faculty debriefed the experience with students, modeling effective communication and physical exam skills. Two of these encounters were part of summative multi-station assessments. During summative encounters, faculty assessed student performance but did not provide feedback. Following all clinical skills examination days, students engaged in small group debriefs led by communication skills faculty to discuss cases and make connections the students may not have made individually.

### Study design

We conducted a qualitative study incorporating confidential focus groups with key stakeholders. Participants studied were second-year medical students at the institution. School of Medicine faculty who coached first- and second-year medical students in both the classroom and the clinical skills settings were invited to participate. SPs had played the role of either the male or the female LSP characters, known as Larry Patterson and Linda Paulson (LP). Four focus groups were held; two with students, one with faculty, and one with SPs. The study was approved by the Northwell Health System Institutional Review Board, and all participants provided written consent to participate and to be audio recorded.

### Participants

Based on funding and researcher availability, a random sample of 16 second-year medical students were invited to participate in the focus groups; 15 of the 16 attended the discussion group. The random sample was selected from the 80-student class using a random number generator. All faculty and LSPs who participate in clinical skills teaching at the School of Medicine were invited to participate in focus groups. All eight faculty and 10 of 16 SPs agreed to participate.

### Focus groups

We used focus groups to gain insight into medical student, SP and faculty perceptions, beliefs and experiences with the LSP program. We conducted separate homogenous focus groups with SPs, faculty, and medical students to encourage authentic and unique reflections of their experience with the LSP program. Each focus group lasted 60–90 min and included 7–10 participants. An experienced qualitative researcher not responsible for evaluation of participants led the focus groups.

Focus group discussions concentrated on continuity, relationships, and feedback (see moderator guide in Appendix Table A1). All focus group questions were pilot tested with three third-year medical students and two faculty members not included in the focus groups to optimize face and content validity.

The leader asked all participants to answer the introductory question about overall perceptions of the clinical skills experience. Participants chose whether to answer subsequent questions. Key questions involved perceptions of continuity, relationships, and feedback and were posed in the same order in all focus groups. The leader moved from one question to another when saturation was reached or no new concepts were being generated after repeated probing. The closing question in each group asked how participants would change the program to improve the LSP experience. Focus group proceedings were audio recorded and professionally transcribed. We identified and analyzed relevant themes and quotes using a template analysis approach [14]. No compensation other than snacks or light lunch was offered to faculty and student participants. As per institutional policy, SPs were paid at their normal hourly rate for participation.

### Data analysis

With a pragmatist orientation, we analyzed our qualitative data thematically using a template analysis approach described by King in 2004 [14]. Template analysis is a highly flexible analytical approach which allows for a balance in having structure in the analysis process and being open to the data [14]. It is also particularly useful when comparing views from different groups within a specific context, which was a major objective of the current study. [14] An initial template was created with higher-order codes based on key questions in the moderator guide.

Feedback, continuity, and relationship-building were defined a priori and used deductively as the initial template to guide analysis, but were modified, adding new codes and deleting unused codes, changing scope, and changing higher-order classification, based on ongoing inductive analysis approach. Analyst triangulation was conducted by having two researchers (LB and RD) independently code the text and reflect upon relevant themes and quotations, further developing these themes [15]. Triangulation of sources was also conducted by comparing student, faculty, and SP viewpoints [15]. Themes were pooled until saturation was reached. Disputes were resolved by consensus. A third researcher (AK) served as arbiter of disputes, and reviewed the final themes looking for consistency with the original research question. To improve accuracy, validity, and transferability of the results, respondent validation was implemented. Focus group participants were shown a thematic summary and asked whether it conformed to their recall of the focus group sessions. This was done to ensure that the participant meanings and perspectives were accurately represented.

## Results

### Student themes

Students recognized the value of the LSP curriculum to practice clinical skills and receive feedback in a safe environment (Table 2). One student stated that the experience was like, ‘a playground for you to try to do things that either you see your preceptors doing or you see them doing wrong that you think you can do better.’

**Table 2.** Student focus group themes and quotations^a^.

Opportunity to practice and get feedback in safe environment	‘A playground for you to try to do things that either you see your preceptors doing or you see them doing wrong that you think you can do better.’ ‘Putting yourself out there, making mistakes makes you more comfortable and confident in the real situation and I think it’s something just subconscious.’
Feedback was valuable	‘It’s really valuable when we had feedback from the SP and a faculty mentor in the room at the time.’
Growth and development over time at clinical skills	‘track your progress in terms of your skills and your Knowledge about medicine and how to treat patients.’
Tension between formative feedback and summative assessment	‘Difficulty in using [clinical skills] as a tool for assessment rather than as a playground for us to practice learning skills that we don’t really have a safe space to develop in any other place.’
Feedback could be more personal and thoughtful	‘It sounded like [the faculty] were using buzzwords, for example you maintained really good eye contact, [which were] just very basic.’ ‘Patients sometimes guard the information and I feel like while I do an okay job interviewing them I have to ask a specific combination of words in a question to elicit the answer to the scenario.’
Over time, relationship with the female character evolved	‘It really helps with that patient relationship that you remember who they are and remember the past history.’
Male SP case could be more realistic	‘They were all different heights and weights and ethnicities. It was just completely different and it was kind of hard to start the conversation as a friend.’

^a^Themes and representative quotations from focus groups with students participating in a longitudinal standardized patient experience.

Student comments related clinical skills to their own growth and development. One student said, ‘Putting yourself out there, making mistakes makes you more comfortable and confident in the real situation.’ Another student said longitudinal cases allowed students to, ‘track your progress in terms of your skills and your knowledge about medicine and how to treat patients.’

Students noted that LSP cases offered the opportunity to receive individualized, learner-centered feedback on difficult communication skills. As one student said, ‘it’s really valuable when we had feedback from the SP and a faculty mentor in the room at the time.’ Students noted that the longitudinal nature of feedback was helpful, particularly when given by the same preceptor or SP, ‘you can see the progress you’re kind of going through and it can really guide you in the right direction.’

### Relationship-building

Students noted that over time, their relationship with the character evolved, particularly in the female LSP case. As one student said, ‘It really helps with that patient relationship that you remember who they are and remember the past history.’ Several encounters with the female LSP triggered emotional reactions among the students. One student said, ‘we were discussing these emotional things and she would have an emotional reaction and I remember leaving the breaking bad news session and feeling really, really bad for her.’ Another student said, ‘I was there for several of the important events for [Linda’s] life I do feel like there was a relationship because of that.’ Students recognized that this emotional connection constituted relationship-building. One student noted, ‘We were really practicing developing a relationship with a patient and talking about social aspects of their lives, not necessarily medical aspects.’ Students felt that when the cases were at their best, students were able to suspend disbelief and treat the patient like a ‘real’ patient, ‘a few minutes into the encounter [I] kind of forget that it’s not real and really end up treating the patient as if that’s a complaint that they really have. And I think that’s very valuable.’

On the other hand, students found male SP (Larry) case limited relationship formation. Rather than evolving illnesses, students perceived that they encountered isolated symptoms and conditions in the encounters with Larry. One student noted, ‘he has a new disease each time that you specifically need to address.’ Another student noted, ‘he’d come back six months later with a new problem completely unrelated to his first and second problem.’ A third student felt that the case ‘feels very staccato and doesn’t really flow at all.’

Having different actors, particularly actors of different ages and ethnicities, was felt by students to limit relationship-building. One student noted the patients ‘were all different heights and weights and ethnicities. It was just completely different and it was kind of hard to start the conversation as a friend.’ Students also noted that the SPs did not make an attempt to remember the students, which seemed to students like a missed opportunity. One student noted, ‘They never reference back to any previous encounters.’ Another student noted, ‘They never remember your performance from the previous time. They can’t comment on it. So, what’s the point of having the same person?’

Several students also noted that occasionally, students and SPs would recognize each other from prior encounters. As one student recalled,

when I saw her again … I could tell she recognized me. Obviously I recognized her and I felt familiarity with her [but] because it was supposed to be a new patient, it was supposed to be completely different. And so, that can be a little bit jarring.

Students recommended maintaining continuity with the same patient and faculty member would have enhanced the value of the experience. One student commented, ‘seeing the same person really helps.’ Several students recommended that where the LSP could not be the same person, keeping students assigned to SPs of same general age or ethnicity would aid in authenticity. As students hoped to use the LSP cases to follow diseases as well as patients, seeing the evolution of a chronic disease through the eyes of the LSP would have been helpful, such as ‘if we had followed the progression the disease say like liver disease from fatty liver to cirrhosis.’

Students noted the long time between visits and asked for a stronger chart to facilitate recall, stating ‘And part of that I think has to do with having charts on last times so we’re familiar with what happened and why that would be connected to what’s going on now.’ Another student commented, ‘I think we should be given the opportunity to look over the chart a little bit longer before going into the room because that’s how things are in real life.’

### SP themes

SPs saw their role as an important one in medical student education (Table 3). One SP noted, ‘It meant so much to see someone go from slouchy, discouraged, to now feel like, “I have this. If I’m ever in this situation, I know where to go with this.”’ The experience of guiding students through difficult experiences was for empowering the SPs. An SP noted, ‘this person [student] who was sent to take care of me – the role was reversed. She needed someone to take care of her. So it was very interesting to see how quick that changed for her.’

**Table 3.** SP focus group themes and quotations^a^.

Role as an important in medical student education	‘It meant so much to see someone go from slouchy, discouraged, to now feel like, “I have this.” If I’m ever in this situation, I know where to go with this.’
Instilling empathy	‘We’re instilling humanity in it before med school rips it out.’
A safe learning environment for real-time feedback	‘We were debriefing, I really chose my words carefully, and he ended the debrief with, ‘That was the most constructive conversation I’ve ever had, and I’m going to take this with me for the rest of my life.’'
Students genuine and authentic during their encounters	‘The learner is sitting there listening to your tale of woe, so to speak, and you can see the empathy in their face, and how difficult it is. And again, it is. It’s real life. People go through these situations all the time.’
SPs had the impression that students remembered them over time	‘Even if it wasn’t the student that I literally had that last time, they remembered [the character] and said, “How are you?” You know, “Are you still excited about the baby?” And that showed me something.’
SPs felt they were more invested in LSP cases than other cases	‘Something that I found interesting when I first began at SP here is everybody was talking about Linda and Larry, and I thought they were real people.’
Limitations to the continuity, integrity, and fidelity of the LP cases	‘We had to push in that we were a veteran out of nowhere. It had nothing to do with anything of the case at all.’
Sometimes students would confuse cases	‘Sometimes they remember you from past cases, and they’ll literally tell about details from other cases – like, you kind of have to give them a look.’

^a^Themes and representative quotations from focus groups with standardized patients participating in a longitudinal standardized patient experience.

SPs perceived the students as being genuine and authentic during their encounters. An SP related,

the learner is sitting there listening to your tale of woe, so to speak, and you can see the empathy in their face, and how difficult it is. And again, it is. It’s real life. People go through these situations all the time.

Another SP said, ‘The more you know your case … and the more real you make it, the more real of a situation it is for them, and it helps them to buy in immediately.’

The SPs took pride in filling a distinct role from faculty in educating students. SPs saw their role as maintaining empathy during medical student education, ‘We’re instilling humanity in it before med school rips it out.’ SPs felt a connection with their longitudinal cases and characters. One SP noted, ‘Something that I found interesting when I first began as an SP here is everybody was talking about Linda and Larry, and I thought they were real people.’ SPs noted feeling more invested in the Linda and Larry cases than other cases. One SP said, ‘oftentimes you grow a liking to the character you’re going to portray, and you’re like push-pulling for them, you know?’

SPs saw relationships develop between the students and the LSP characters despite the lack of continuity. An SP remembered, ‘Even if it wasn’t the student that I literally had that last time, they remembered [the character] and said, “How are you?” You know, “Are you still excited about the baby?” And that showed me something.’ Another SP related,

I have seen students come in and say, “Oh, it’s so good to see you again,” they know that – they’re pretending that, but then when they get into the conversation, it kind of starts to feel a little more real.

Another SP related that students appreciated having an ongoing story line, ‘They’re connected to the character, but not necessarily the patient that’s presenting the character.’

Despite the limitations of the cases, SPs felt it was,

pretty amazing to have that kind of patient where they can see them multiple times. And it’s really mimicking a general practice, just really building that rapport with a patient, and learning them, and remembering all of these things in their lives so they can come at it from one encounter to the next.

SPs took their role in providing feedback very seriously, noting, ‘it’s a safe learning environment, and we let her know that from the beginning.’ Another SP said, ‘When we were debriefing, I really chose my words carefully, and he ended the debrief with, ‘That was the most constructive conversation I’ve ever had, and I’m going to take this with me for the rest of my life.’ SPs knew that as opposed to real practice, students in the clinical skills lab had a rare opportunity to re-practice skills, stating,

that they get an opportunity to immediately get feedback, and then be able to redo something. Because in their practice, they’re not going to get a chance to do that. And it gets so it’s just learning, it’s doing, it’s being able to correct, and I particularly like being a part of that.

Similarly to students, SPs recommended preserving continuity with students to improve the fidelity of the cases. One SP told the group, ‘I don’t think I’ve ever had the same students.’ Another SP noted more positively, ‘I think that would be pretty amazing to give them a little taste of really building relationship with someone.’

Preserving continuity, noted the SPs, would also mean limiting student contact with that SP for other cases. Like the students, SPs related that sometimes students would confuse various cases since the SPs were the same, limiting authenticity of the case. To redirect students, one SP said, he broke character, ‘they’ll literally tell about details from other cases – like, you kind of have to give them a look.’ Another SP noted having signals to let a student know that they were confusing details from a separate case, ‘and it becomes a little bit of a smirk here, a wink there, you know?’

Like the students, SPs recommended stronger charts to assist in recall of details for the students. One SP noted, ‘It’s a skill that you need to read the chart before you walk into a room, for any patient. But especially one that you’re seeing over the course of years.’ Another SP recommended having the students reference their own evolving chart on the patient, ‘But not even a door chart, but like their own notes, like with the – that they take with them, and really kind of absorb.’

### Faculty themes

Similar to SPs and students, faculty observed students grow and develop clinical skills over time through SP experiences (Table 4). They saw students become more fluent in clinical reasoning, communication and physical exam skills, ‘I think they’re more comfortable touching the patient.’ Another faculty noted, ‘they become much more intuitive in terms of making assumptions that we all probably make.’

**Table 4.** Faculty focus group themes and quotations^a^.

Learn and develop clinical skills over time through SP experiences	‘I think they’re more comfortable touching the patient.’ ‘they become much more intuitive in terms of making assumptions that we all probably make.’
Feedback in clinical skills is a formative experience in which students incorporate skills learned into practice, which is enhanced by having the same faculty–student pairing	‘It becomes more of a quality experience when you have the same student that you follow, because you do get to see them grow, and you see that they took in your feedback and applied it.’
LSP model provided students with the potential for relationship-building	‘To recollect what happened at the last visit and sort of build on that’
Continuity pairings of students and LPs facilitated continuity	‘Does give them the feeling of continuity.’ ‘see the difference in comfort level with LP if they’ve seen them [the SP] before.’
Personal satisfaction watching students grow over time	‘I take pride in the fact that I … help them form to what they are now.’
Assessment sometimes precluded the opportunity to mentor students	‘It’s very clear that people who evaluate somebody, it’s very hard to be a mentor.’

^a^Themes and representative quotations from focus groups with faculty participating in a longitudinal standardized patient experience.

For the faculty, watching this growth was personally satisfying. One faculty member noted, ‘What I love is watching them outside the room, being able to identify some of the skills that we’ve taught in session and they’re struggling with, and some of the skills they’re doing great with both.’ The continuity of the LSP cases allowed faculty to accelerate this growth, as one faculty said,

it’s great to see the progression from starting very early in their career to mature medical students, and to see them taking care of real patients and doing real procedures. I take pride in the fact that I … help them form to what they are now.

Another faculty member noted,

I saw a student in do a physical. I would say it’s about two months ago. And there were some particular things that we had talked about in terms of communication skills … I saw her put that into practice today and it was very effective.

Faculty noted the LSP model allowed students, ‘to recollect what happened at the last visit and sort of build on that’ and noted the experience, ‘does give them the feeling of continuity.’ Faculty noted that longitudinal pairings of students and LPs facilitated this continuity, explaining, you can ‘see the difference in comfort level with LP if they’ve seen them [the SP] before.’ Another faculty noted,

I have seen a student have the same actor or actress for LP, and you could even see the difference in comfort level with LP if they’ve seen them before. You could just see from the interaction that … when it is the same actor or actress, it makes a difference.

On the other hand, faculty noted that when continuity pairings were lacking, ‘individual interactions might not be as, I guess, as seamless as the case, because of the fact that they’re dealing with different age of SP, and a different look, you know, different body.’

Faculty noted that feedback in clinical skills is a formative experience in which students incorporate skills learned into practice, which is enhanced by having the same faculty–student pairing. As one faculty noted, ‘It becomes more of a quality experience when you have the same student that you follow, because you do get to see them grow, and you see that they took in your feedback and applied it.’ Another faculty noted how continuity with faculty is a source of relief for student, ‘I also think the students feel relieved that the faculty is very familiar to them. So many times I’ve walked into the [room] nervous, and they see me, and they just kind of relax.’

Like students and SPs, faculty noted that sustaining SP–student–faculty pairings would improve the authenticity of the cases and individualize feedback. One faculty noted the benefit of having the same students repeatedly in LSP encounters, ‘Well it allows us to really connect with them. If this person was seen before, these are certain things that would be more appropriate to do or not do, depending on what happened.’

Faculty felt an individualized door chart created by the students would facilitate recall and the authenticity of the case. Just as physicians make notes to assist their recall of patients, students should create notes on the LSP case, ‘cause in the real world, you take a chart out, you know you’ve seen that patient before, because you have your notes and different things.’ Another faculty member suggested having students, ‘Write something that’s going to help you remember this patient for the next visit.’

Faculty noted the tension between assessing and mentoring students. One faculty said, ‘It’s very clear that people who evaluate somebody, it’s very hard to be a mentor, because right away they’re looking to please.’

## Discussion

The main objective of our study was to characterize the nature of relationship-building, feedback, and continuity among all stakeholders participating in a single LSP program. Our findings show both similar and divergent perspectives between the stakeholders regarding the LSP program. An LSP experience for medical students introduced the importance of continuity of care to students, the key role of faculty and SPs in providing feedback, and the opportunity for relationship-building. While all stakeholders saw growth in student skills and the benefits of simulating a physician–patient relationship, faculty, students, and SPs recognized that lack of continuity pairings limited its effectiveness.

Stakeholders observed that the LSP cases brought some sense of continuity missing in other clinical skills encounters which helped prepare students for patient care. The SPs noted feeling wedded to the characters to a greater extent than other cases. The opportunity for relationship-building and feedback through longitudinal cases was felt to be incompletely realized due to a lack of continuity of student–faculty–SP pairings. Students, SPs, and faculty recommended matching students and actors across encounters when feasible to, ‘help build that rapport, maintain that rapport, and make it feel like a real patient.’ Where not possible to match students and SPs, it was recommended to ensure students see SPs of similar ages and ethnicities.

Students also recommended that having the SPs note a key detail about student performance would enhance feedback and relationship-building. SPs who participated in an LSP program in the Netherlands involving continuity pairings noted an opportunity for individualized feedback [10].

Students and faculty noted the tension between formative feedback and summative assessment inherent in the LSP program. Medical educators have noted the value of summative feedback in improving knowledge and changing behavior by encouraging preparation, and in letting students know where they stand relative to peers. The value of formative assessment includes shaping skills and knowledge through modeling and coaching, and helping students forge a professional identity in which reflection and personal growth are valued [16]. A clinical skills environment facilitates the achievement of both of these aims, but may be best accomplished if these assessments are carried out by different individuals, such as coaches versus evaluators.

Members of all groups recommended door charts as a way to facilitate recognition of cases over time and allow students to build skills in documentation. All stakeholders recommended using a chart to facilitate recall and authenticity of the case. The challenge of remembering patients previously seen months or years previously is one faced by doctors in outpatient practice and facilitated by charts, which can easily be brought to the clinical skills arena and in which students can contribute their notes on patients [17].

The inclusion of multiple stakeholder groups was a strength of this study, given convergent themes around student growth, feedback, and relationship-building. The SP is an important source of feedback in clinical skills and objective structured clinical examination experiences. The SP perspective should be considered in further studies involving clinical skills environments.

Limitations of this study include a single institution, and results may not be transferable to other institutions. As a small sample of students and just over half of SPs participated in focus groups, not all voices may have been heard. Faculty and SP time constraints limited faculty–SP–student pairings, which impacted relationship-building and feedback.

An LSP experience for early medical students was viewed by students, faculty, and SPs as a way to enhance the experience of continuity in clinical skills. Several key lessons were learned by key stakeholders from the longitudinal experience. The experience was successful in wedding SPs to their characters and providing a mechanism for graded coaching and feedback by SPs and faculty. Lack of continuity pairing among students, SPs, and faculty was seen as a barrier, limiting mentorship and case authenticity. Continuity pairings were encouraged by students, faculty, and SPs to strengthen the experience. Similarly, as SPs also played distinct characters encountered by medical students in clinical skills, students and SPs pointed out it would be preferable to avoid having students encounter the SPs from longitudinal cases in other scenarios. Key recommendations from stakeholders in building an LSP program include the value of individualized door charts to prompt case recall by students, the importance of individualizing feedback by SPs, and the need to emphasize the faculty role in feedback and coaching rather than assessment.

## Appendix 1. Focus group questions

**Table:**

Students
What is most memorable to you about clinical skills?
What has been your overall experience seeing Larry Patterson and Linda Paulson in clinical skills over the past 2 years?
In what ways did Linda and Larry feel like real patients?
In what ways did Linda and Larry differ from real patients?
Has seeing Linda and Larry been different than seeing other SPs? If so, how? If not, why not?
Did you encounter the same Linda and Larry SP repeatedly?
If not, did seeing Linda and Larry feel like the same patient from encounter to encounter?
If not, what could be done differently to make it feel like the same patient from the previous encounter?
Do you feel you have a relationship with LP? If so, describe that relationship? If not, why do you not feel you have a relationship with LP?
How does encountering Linda and Larry repeatedly over 2 years impact your relationship with Linda and Larry?
Describe any feedback or mentoring you have received from faculty or SPs based on your interactions with Linda and Larry.
How has this feedback changed your patient interactions in the initial clinical experience?
How would you change the Linda and Larry program to improve the learning experience?
*Faculty*
What has been the most memorable part of being core faculty in clinical skills?
What has been your experience providing feedback to students as they saw Larry Patterson and Linda Paulson in clinical skills over the past 2 years?
In what way does the Linda and Larry program simulate real patient care experiences?
What limitations does the LP program have in simulating real patient care?
How do student interactions with LP change over the first 2 years of medical school?
Have you seen professional relationships develop between the students and Linda and Larry? If so, describe these relationships. If not, why do you feel a professional relationship has not developed between students and Linda and Larry?
How do the students respond differently to Linda and Larry than to other SP characters?
Has there been any feedback you have provided to students based on the Linda and Larry program that has been particularly important or meaningful to you?
How does the Linda and Larry program impact your feedback and mentoring?
How would you change the Linda and Larry program to improve student learning?
*SP*
What has been the most memorable part of being a standardized patient?
What has been your experience with Larry Patterson or Linda Paulson in clinical skills over the past 2 years?
How does it feel to be Linda or Larry?
How does your experience being Linda or Larry differ from your experience being other characters?
Describe the relationship that the students develop with Linda or Larry.
How do the students respond differently to Linda and Larry than other SP characters?
How have your experiences been different when you encountered the same students multiple times as Linda or Larry?
Describe the feedback you have provided feedback to students in your role as Linda or Larry.
How does the Linda and Larry program impact your ability to provide feedback?
How would you change or improve the Linda and Larry character or program?
